# Clozapine treatment of a Japanese patient during pregnancy: Effect on fetal heart rate

**DOI:** 10.1002/npr2.12486

**Published:** 2024-09-16

**Authors:** Shunya Aoki, Katsutoshi Takada, Tatsuru Sugama, Mitsugi Kimiwada, Tatsuya Hoshino, Takaoki Kaneko, Shintaro Obata, Yasuhiro Ota, Satoshi Toishi, Kaori Koike, Hirokazu Akada, Takahisa Saiga, Shigeki Sato

**Affiliations:** ^1^ Pharmaceutical Department Japanese Red Cross Narita Hospital Narita‐shi Chiba Japan; ^2^ Nursing Department Japanese Red Cross Narita Hospital Narita‐shi Chiba Japan; ^3^ Department of Obstetrics and Gynecology Japanese Red Cross Narita Hospital Narita‐shi Chiba Japan; ^4^ Department of Neonatology Japanese Red Cross Narita Hospital Chiba Japan; ^5^ Department of Psychiatry Japanese Red Cross Narita Hospital Narita‐shi Chiba Japan

**Keywords:** clozapine, drug monitoring, Japanese people, maternal‐fetal exchange, pregnancy

## Abstract

The current literature on the effects of clozapine on pregnancy is limited, and no cases of pregnant Japanese women have been reported. Decreased variability in the fetal heart rate due to clozapine exposure has been reported in countries other than Japan, but its association with serum concentrations of clozapine has not been documented. In this case, a 29‐year‐old Japanese primipara with treatment‐resistant schizophrenia taking clozapine 250 mg/day experienced pregnancy. The pregnancy progressed without complications. At 40 weeks and 2 days of gestation, the patient developed premature rupture of membranes, and decreased variability in the fetal heart rate and variable deceleration were observed, leading to an emergency cesarean section. The neonate had no congenital malformations, metabolic disorders, seizures, floppy infant syndrome, leukopenia, or neutropenia. Serum concentrations of clozapine and norclozapine (N‐desmethylclozapine), measured in the mother and in the neonate immediately after birth, suggested that clozapine and norclozapine were transported to the fetus during pregnancy. Based on these observations, the present case suggests that high fetal serum concentrations of clozapine and norclozapine may affect fetal heart rate. This case report concludes that, with careful monitoring, Japanese women taking clozapine can deliver successfully and emphasizes the importance of monitoring serum clozapine concentrations and fetal cardiac function throughout pregnancy, with particular attention to the later stages.

## Abstract 2

Japanese pregnant woman on clozapine delivered successfully. High fetal serum concentrations of clozapine and norclozapine may affect fetal heart rate. Monitoring clozapine serum concentrations and fetal cardiac function is crucial for safe pregnancy.

## Graphical abstract

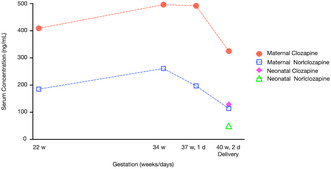

## 1 INTRODUCTION

Clozapine is the only atypical antipsychotic agent indicated for treatment‐resistant schizophrenia. According to a recent systematic literature review involving 42 pregnant women exposed to clozapine, clozapine does not increase the risk of teratogenicity, stillbirth, miscarriage, fetal abnormalities, delivery complications, or preterm birth. Clozapine has a high potential for transfer into breast milk. However, cases of clozapine use during pregnancy are limited, and evidence regarding the effects of clozapine on the mother and child is insufficient.^1^

In particular, no cases of Japanese women taking clozapine during pregnancy have been reported. In countries other than Japan, the influence of fetal clozapine exposure on fetal heart rate (FHR) has been reported,^2^, ^3^, ^4^, ^5^, ^6^ including decreased FHR variability,^2^, ^3^, ^4^ absence of FHR reactivity,^5^ and abnormal FHR.^6^ However, the relationship between serum clozapine concentrations and variations in FHR remains unclear.

This case report describes a pregnant Japanese woman with treatment‐resistant schizophrenia who took clozapine and gave birth, along with the clozapine and norclozapine (N‐desmethylclozapine) serum concentrations in the mother and neonate.

Written consent for publication was obtained from the participant.

## 2 CASE

A 29‐year‐old Japanese primipara with treatment‐resistant schizophrenia had a history of alopecia. The patient had no history of smoking and demonstrated strong adherence to the prescribed medication regimen. Her pre‐pregnancy body mass index was 20.5 kg/m^2^. She developed schizophrenia with hallucinatory and delusional states at age 24. She was diagnosed with treatment‐resistant schizophrenia at age 25 because she did not respond to multiple atypical antipsychotics. Subsequently, she was started on clozapine. Her psychiatric symptoms stabilized with clozapine 250 mg/day, and she was followed up as an outpatient.

At age 29, she had an unplanned pregnancy, discovered at 9 weeks and 2 days of gestation. During pregnancy discovery, her medications included clozapine 250 mg/day, magnesium oxide 660 mg/day, and midodrine 4 mg/day, but midodrine was immediately discontinued. The dose of clozapine was not changed during pregnancy. The pregnancy progressed without complications, without gestational diabetes or hypertension. The patient's psychiatric symptoms remained well controlled with continued clozapine treatment throughout the pregnancy.

**FIGURE 1:**
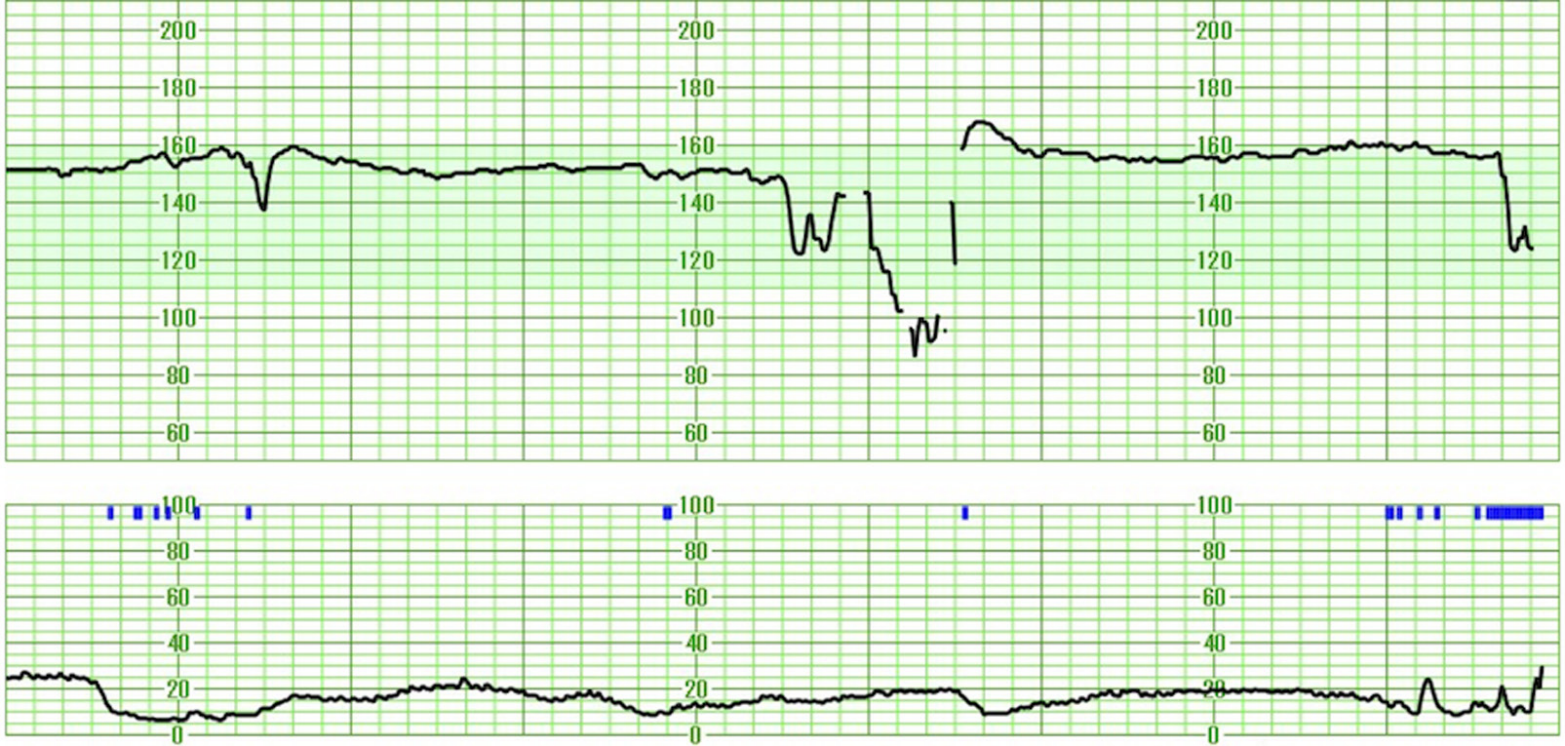
Fetal heart rate (FHR) at 40 weeks and 2 days of gestation in a pregnant Japanese woman taking clozapine 250 mg/day. Cardiotocography showed decreased FHR variability and variable deceleration.

At 40 weeks and 2 days of gestation, she was admitted for premature rupture of membranes. Cardiotocography showed decreased FHR variability and variable deceleration (Figure 1), leading to an emergency cesarean section for fetal dysfunction. There were no intraoperative or postoperative complications. The mother did not experience any physical or psychological complications postpartum.

A female neonate weighing 3010 g, measuring 49 cm in length, and with a head circumference of 33.5 cm was delivered. The Apgar scores were 8 and 9 at 1 and 5 min, respectively. The post‐delivery umbilical artery pH was 7.297, indicating no fetal acidosis. The neonate had no congenital malformations, metabolic disorders, seizures, or floppy infant syndrome. The neonate's white blood cell count was 15.3 × 10^9^/L and neutrophil count was 7.3 × 10^9^/L, without leukopenia or neutropenia. The neonate was monitored in the neonatal intensive care unit for 1 day as a precautionary measure, but no abnormal findings were observed.

**FIGURE 2:**
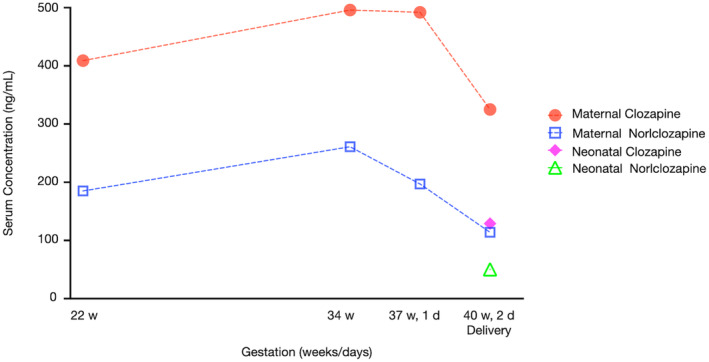
Maternal clozapine and norclozapine (N‐desmethylclozapine) serum concentrations in a pregnant Japanese woman taking clozapine 250 mg/day and clozapine and norclozapine serum concentrations in the neonate exposed to clozapine. In the x‐axis label, w represents weeks, and d represents days. The detailed values of clozapine and norclozapine in the serum are listed in Table S1.

Changes in clozapine and norclozapine serum concentrations in the mother during the second and third trimesters and at delivery, as well as in the neonate immediately after birth, are shown in Figure 2. The detailed clozapine and norclozapine serum values are shown in Table S1; the serum concentrations before pregnancy and during the first trimester could not be measured because of inadequate measurement technology at our hospital.

The mother fed the infant with formula and avoided cabergoline for lactation suppression.

## 3 DISCUSSION

This is the first case report of a Japanese woman with treatment‐resistant schizophrenia who conceived and gave birth while taking clozapine. Measurements of clozapine and norclozapine serum concentrations in the mother and neonate immediately after birth revealed that both substances were transported to the fetus during pregnancy and suggested that fetal exposure to clozapine affects FHR, particularly when maternal and fetal serum concentrations of these substances are high.

First, this case highlights that clozapine can be successfully administered to pregnant Japanese women. In Japan, clozapine was approved in 2009, later than in other countries, and its prescription rate is lower.^7^ Consequently, no cases of pregnant Japanese women taking clozapine have been reported. Furthermore, the main metabolic enzyme of clozapine is cytochrome P450 (CYP) 1A2, and the proportion of Japanese individuals with decreased CYP1A2 activity is higher than in other populations.^8^ Because pharmacokinetics differ between ethnicities, reports of clozapine use during pregnancy and delivery in different populations are important. In countries other than Japan, previous studies on the effects of clozapine during pregnancy have reported gestational diabetes^9^ and hypertension^10^ in mothers and malformations,^11^ seizures,^12^ floppy infant syndrome,^13^ and neutropenia^14^ in fetuses, but none were observed in this case. Clozapine concentrations in the maternal serum are influenced by estrogen levels, which progressively increase toward the end of pregnancy.^15^ Estrogen inhibits CYP1A2, resulting in elevated clozapine concentrations in the maternal serum.^8^ In this case, as in previous reports, serum clozapine concentrations gradually increased as the pregnancy progressed, which may be attributed to CYP1A2 inhibition associated with increasing estrogen levels throughout pregnancy. The decrease in clozapine concentration in the maternal serum on the day of delivery is supposedly due to intravenous fluid administration associated with the cesarean section. Regarding breastfeeding, the mother used formula feeding and avoided cabergoline. Although the relative infant dose of clozapine is 1.33%–1.4%,^16^ agranulocytosis and sedation in infants due to exposure to clozapine through breastmilk have been reported.^17^ Therefore, the potential risks and benefits of breastfeeding while taking clozapine should be carefully considered. Similarly, cabergoline for lactation suppression should be carefully used in patients with schizophrenia, as it is associated with worsening psychiatric symptoms.^18^

Second, this case suggests that fetal exposure to clozapine may affect FHR. In this case, we observed decreased FHR variability and variable decelerations. Generally, FHR abnormalities are used to diagnose fetal dysfunction, with the severity of abnormalities indicating the degree of dysfunction, although they frequently generate false‐positive results.^19^ When decreased FHR variability and variable deceleration are observed, they warrant careful attention, as these changes may signify compromised fetal well‐being and potentially lead to fetal asphyxia if not properly managed.^20^ Decreased FHR variability can present in cases of fetal hypoxia or acidosis in utero, fetal nonrapid eye movement (REM) sleep state, or immaturity.^21^ Additionally, central nervous system depressants, including anesthetics and phenothiazines, can cause decreased FHR variability by affecting the autonomic nervous system.^21^ Variable decelerations, commonly observed patterns in FHR monitoring, typically result from vagal reflexes triggered by blood pressure fluctuations due to umbilical cord compression during uterine contractions, leading to temporary interruption of umbilical blood flow, or by fetal hypoxia or acidosis.^21^ Moreover, the secondary occurrence of variable decelerations is reportedly a consequence of drug exposure.^22^ In this case, other potential causes of decreased FHR variability and variable decelerations, apart from clozapine exposure, were systematically evaluated and excluded. Fetal asphyxia and acidosis were excluded based on the post‐delivery umbilical artery pH of 7.297 and Apgar scores of 8 and 9 at 1 and 5 min, respectively. The possibility of non‐REM sleep states causing these FHR patterns was discounted because the patterns persisted throughout labor, extending beyond the typical duration of these states, which rarely last more than an hour during active labor. Additionally, fetal immaturity was precluded as a contributing factor due to the gestational age of 40 weeks and 2 days and the absence of fetal malformations. Therefore, clozapine exposure is practically the most significant factor affecting FHR in this case.

Clozapine reportedly crosses the blood–placental barrier and is exposed to the fetus.^1^ Several reports have described that fetal clozapine exposure influences FHR,^2^, ^3^, ^4^, ^5^, ^6^ suggesting that clozapine and its main metabolite, norclozapine, may affect FHR. Clozapine blocks α1‐adrenergic and muscarinic receptors in the myocardium, causing bradycardia in adults, electrocardiogram changes, and decreased heart rate variability.^23^ Additionally, norclozapine potentially contributes to peripheral adverse drug reactions such as cardiovascular dysfunction, constipation, or hypersalivation in adults.^8^ Moreover, it can be transported from the mother rather than being derived from fetal metabolism.^1^ In this case, as observed in adults, clozapine and norclozapine exposure may have affected autonomic function, leading to decreased FHR variability and variable deceleration in the fetus.

Therefore, serum concentrations of clozapine and norclozapine could be useful to predict effects on FHR, but the relationship between serum concentrations of clozapine and norclozapine and various adverse effects during pregnancy, including effects on FHR, remains largely unclear. Previous reports of clozapine's effects on FHR involved doses ranging from 100 mg to 400 mg, but essentially, serum concentrations of clozapine and norclozapine were not measured.^2^, ^3^, ^6^ Conversely, the highest reported maternal clozapine dose at delivery was 350 mg/day, with a corresponding serum clozapine concentration of 77 ng/mL, and no effects on FHR were observed.^9^, ^24^ Importantly, in this case, a clozapine dose of 250 mg/day resulted in a maternal serum clozapine concentration of 129 ng/mL at delivery, which was higher than those reported in cases without FHR effects.^9^, ^24^ The higher clozapine concentration despite a lower dose might be attributed to decreased CYP1A2 activity, which is more common in Japanese individuals,^8^ leading to delayed clozapine metabolism. This metabolic delay may have resulted in increased fetal transport of both clozapine and norclozapine, potentially leading to effects on FHR. Neonatal serum concentrations immediately after birth have been documented, ranging from 27 ng/mL to 113 ng/mL and 26 ng/mL to 56 ng/mL for clozapine and norclozapine, respectively, with no reported effects on FHR.^9^, ^24^ In this case, the neonatal serum concentrations were 129 ng/mL for clozapine and 50.4 ng/mL for norclozapine, which were higher than those previously reported, particularly for clozapine. The higher clozapine concentrations at delivery in the mother and neonate in this case compared to previous reports may have contributed to the observed decreased FHR variability and variable deceleration. Alternatively, considering that FHR effects were observed despite a decrease in clozapine and norclozapine serum concentrations after 37 weeks and 1 day of gestation, and as a previous study reported a relative accumulation of clozapine in the neonate, which was associated with effects on FHR,^24^ the accumulation of clozapine and norclozapine throughout pregnancy may have been a contributing factor affecting FHR. To minimize the potential impact on FHR while maintaining therapeutic efficacy, clozapine and norclozapine serum concentrations should be monitored and maintained at the lowest effective level throughout pregnancy. This is particularly important in late pregnancy, when maternal physiological changes such as alterations in hepatic clearance, increased fluid volume, and changes in plasma proteins may affect clozapine metabolism.^1^, ^25^

In conclusion, delivery is possible in a pregnant Japanese woman taking clozapine. The present case also suggests the importance of periodical monitoring of serum concentrations of clozapine and norclozapine and fetal cardiac function throughout pregnancy, with particular emphasis on the later stages, in the management of pregnant women taking clozapine. As this is a single case report, additional cases are needed to draw definitive conclusions. Further investigation is required to determine whether clozapine's effects on FHR result from relative accumulation of these substances throughout pregnancy, occur transiently near delivery, or are dose‐dependent. Additionally, studies on the long‐term prognosis of infants exposed to clozapine are even more limited^1^ and require further investigation.

## AUTHOR CONTRIBUTIONS

Shunya Aoki, Katsutoshi Takada, Kaori Koike, and Shigeki Sato wrote the manuscript. Shunya Aoki, Takaoki Kaneko, Yasuhiro Ota, and Shigeki Sato treated the mother or the neonate. All authors read and approved the final manuscript.

## FUNDING INFORMATION

The authors declare no funding.

## CONFLICT OF INTEREST STATEMENT

The authors declare no conflict of interest.

## ETHICS STATEMENT

Approval of the Research Protocol by an Institutional Reviewer Board: This case report has been approved by the Ethics Committee of the Institution (Committee of Japanese Red Cross Narita Hospital; approval no. 885–02) and conforms to the provisions of the Declaration of Helsinki.

Informed consent: Informed consent was obtained from the mother.

Registry and the Registration No. of the study/trial: N/A.

Animal studies: N/A.

## PATIENT CONSENT STATEMENT

Written consent for publication was obtained from the participant.

## Supporting information


Table S1.

## Data Availability

The relevant data are contained within this article and supporting information.
