# Supplementary material for: Clozapine treatment of a Japanese patient during pregnancy: Effect on fetal heart rate
Source: Neuropsychopharmacol Rep. 2024 Sep 16;44(4):852–6. doi: 10.1002/npr2.12486 (PMC11609758; doi:10.1002/npr2.12486)
Supplement: Supplementary file 1 — Table S1. [file NPR2-44-852-s001.docx]

**Supporting Information**

**Table S1.** Maternal and neonatal serum concentrations of clozapine and norclozapine.

| Variables | Gestation (weeks/days) | | | |
| --- | --- | --- | --- | --- |
|  | 22 w | 34 w | 37 w, 1 d | 40 w, 2 d |
|  |  |  |  | Delivery |
| Maternal |  |  |  |  |
| Clozapine | 409 | 496 | 492 | 325 |
| Norclozapine | 185 | 261 | 197 | 114 |
| Neonatal |  |  |  |  |
| Clozapine |  |  |  | 129 |
| Norclozapine |  |  |  | 50.4 |
|  |  |  |  | (ng/mL) |

*Note*: Maternal clozapine and norclozapine (N-desmethylclozapine) serum concentrations in a pregnant Japanese woman taking clozapine 250 mg/day and clozapine and norclozapine serum concentrations in the neonate exposed to clozapine
